# Triad Features of Myocardial Bridging in the Left Anterior Descending Artery: An Atypical Cause of Angina

**DOI:** 10.1016/j.jaccas.2025.104656

**Published:** 2025-08-06

**Authors:** Junbo Ge, Jing Yang

**Affiliations:** Department of Cardiology, Zhongshan Hospital, Fudan University, Shanghai, China

**Keywords:** coronary circulation, intravascular ultrasound, myocardial ischemia

**Figure:**
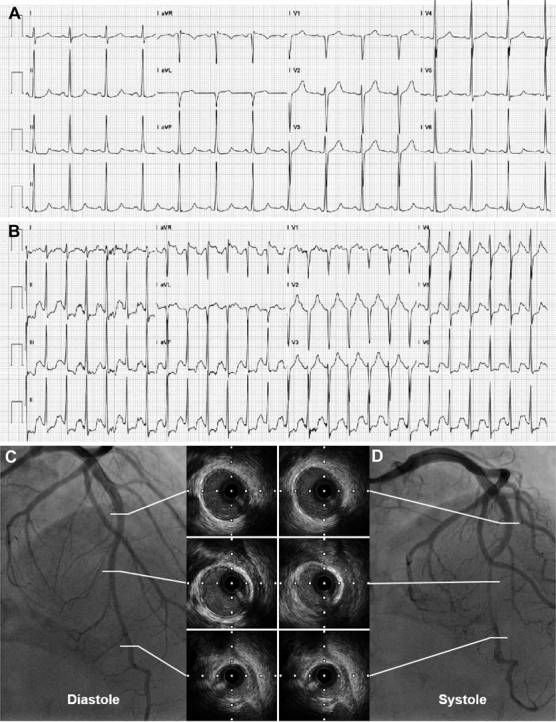

Despite the continuous development of invasive techniques, exercise electrocardiographic stress testing remains the most widely used noninvasive test for detecting signs of myocardial ischemia. One of the clinical manifestations of patients with myocardial bridging (MB) is that the chest pain often lasts much longer than typical angina.^1^^,^^2^

**Take-Home Message**

• Triad features, including unrelieved angina, atypical exercise electrocardiogram, and excessive anxiety may be representative of isolated myocardial bridging in the left anterior descending artery.

We describe new clinical features of a patient with MB who presented with excessive anxiety associated with unrelieved angina and atypical exercise electrocardiogram (ECG) findings. She was diagnosed with coronary artery disease, and her chest pain was not alleviated by the anticoronary artery disease medications; the chest pain was even aggravated after taking a nitroglycerin tablet (500 mg). Physical examination findings, routine resting ECG, and echocardiography results were normal. Cardiac biomarkers including cardiac troponin T and N-terminal pro–B-type natriuretic peptide were also in the normal range. Treadmill exercise ECG was terminated because of moderate angina and significant ST-segment suppression in leads II, III, aVF, and V_4_ to V_6_ (Figures 1A and 1B). The patient was referred for invasive assessments. Coronary angiogram evidenced isolated MB at the left anterior descending coronary artery (LAD) with a typical milking effect; this effect was aggravated by intracoronary nitroglycerin (Figures 1C and 1D). No stenosis was found in other epicardial arteries. Further intravascular ultrasound revealed a half-moon phenomenon at the segment of the milking effect, a typical feature of MB.^1^ These symptoms were relieved by treatment with a ß-blocker (metoprolol succinate 23.75 mg, once daily) and an antianxiety agent (sertraline hydrochloride tablet, 50 mg, once daily).

**Figure 1:**
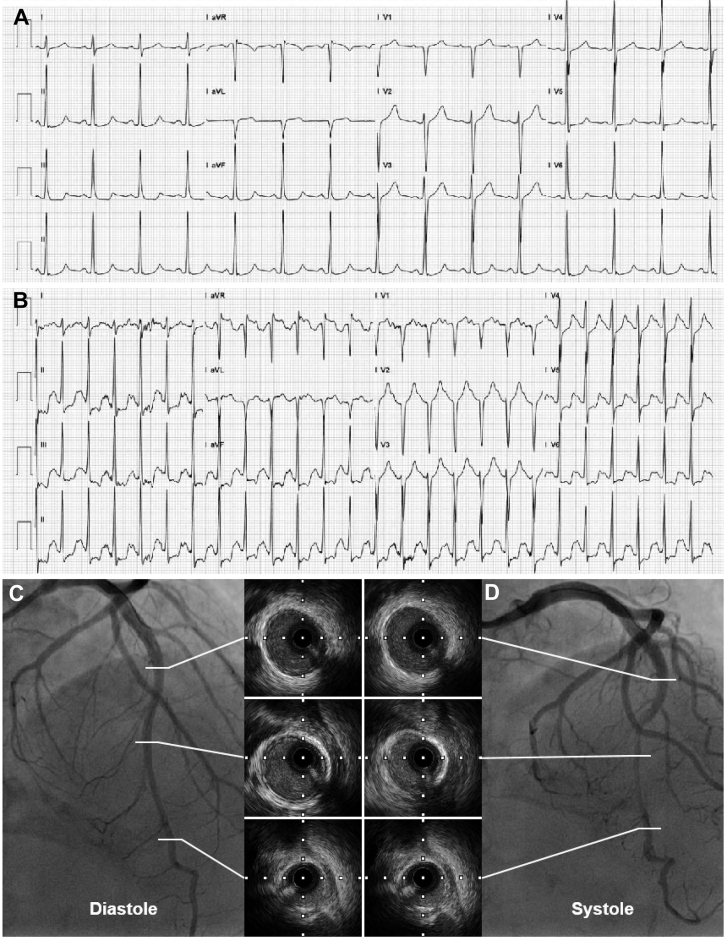
Electrocardiogram, Coronary Angiograms of Left Anterior Descending Coronary Artery, and IVUS Images (A) The 12-lead standard electrocardiogram shows nonspecific ST-segment deviation at rest. (B) Significant ST-segment depression in leads II, III, aVF, and V_4_ to V_6_ during exercise. (C and D) The typical features of coronary angiograms of the left anterior descending coronary artery and IVUS images of the related segments at diastole and systole.

It is known that MB, as a common coronary congenital anomaly, mainly affects the LAD, and can cause ischemia, even myocardial infarction.^2^ Because of the mismatch of the epicardial vessel with MB and the territory of ST-segment suppression during treadmill exercise ECG, we subsequently reviewed our database of MB; to our surprise, a considerable proportion of patients with isolated LAD MB also showed significant ST-segment suppression in leads II, III, aVF, and V_4_ to V_6_ during treadmill exercise ECG. Although the exact mechanism of such exercise ECG pattern remains unclear, possible explanations include compression by myocardial contraction during exercise, reduced coronary flow reserve, and vascular spasm or thrombosis.^3^ Besides, exercise-induced ST-segment depression does not readily provide a reliable assessment of specific coronary vessel involved. The ST-segment depression during exercise stress test in the lateral leads ± the inferior leads in patients with isolated MB might also be unspecific.

## Funding Support and Author Disclosures

This work was supported by the Shanghai Top Priority research center construction project (2022ZZ01010). The authors have reported that they have no relationships relevant to the contents of this paper to disclose.
